# Long-Term Treatment Outcome after Only Popliteal Lymph Node Dissection for Nodal Metastasis in Malignant Melanoma of the Heel: The Only “Interval Node” Dissection Can Be an Adequate Surgical Treatment

**DOI:** 10.1155/2013/259326

**Published:** 2013-05-12

**Authors:** Kentaro Tanaka, Hiroki Mori, Mutsumi Okazaki, Aya Nishizawa, Hiroo Yokozeki

**Affiliations:** ^1^Department of Plastic and Reconstructive Surgery, Graduate School of Medical Sciences, Tokyo Medical and Dental University, 1-5-45 Yushima, Bunkyo-ku, Tokyo 113-8519, Japan; ^2^Department of Dermatology, Graduate School of Medical Sciences, Tokyo Medical and Dental University, 1-5-45 Yushima, Bunkyo-ku, Tokyo 113-8519, Japan

## Abstract

We present a patient with malignant melanoma on his heel. Wide local excision was performed, along with sentinel lymph node biopsy of the inguinal and popliteal lesions. The primary site was clear of tumor at all margins; the inguinal nodes were negative, but the popliteal node was positive for metastatic melanoma. Only radical popliteal lymph node dissection was performed. The patient went on to receive adjuvant chemoimmunotherapy. There was no recurrence or complication until the long-term followup. Popliteal drainage from below the knee is uncommon, and the rate of popliteal-positive and inguinal-negative cases is estimated to be less than 1% of all melanomas. There is no established evidence about how to treat lymph nodes in these cases. Because we considered popliteal nodes as a regional, not interval, lymph node basin, only popliteal lymph node dissection was performed, and good postoperative course was achieved. The first site of drainage is the sentinel node, and the popliteal node can be a sentinel node. The inguinal node is not a sentinel node in all lower extremity melanomas. This case illustrates the importance of individual detailed investigation of lymphatic drainage patterns from foot to inguinal and popliteal nodes.

## 1. Introduction

Sentinel lymph node biopsy is now an essential procedure for the treatment of cutaneous malignant melanoma. Lymphoscintigraphy is commonly used for identification of the sentinel lymph node, which is sometimes detected in unexpected areas, such as the popliteal fossa in distal lower extremity melanoma cases. These are called interval nodes. Popliteal drainage from below the knee is uncommon, and popliteal node metastasis cases are rare. In particular, the rate of popliteal-positive and inguinal-negative cases is estimated to be less than 1% of all melanomas. 

In these cases, management of lymph node dissection is necessary for definitive surgical treatment, but there are no clear answer and no established evidence about how to treat lymph nodes in the literature. Some surgeons do not examine the popliteal fossa, do not perform popliteal node biopsy, or consider the presence of popliteal metastasis positivity as an indication for inguinal dissection.

We present a case of a patient with a malignant melanoma on his left heel, who had positive popliteal node metastasis and negative inguinal node. Only popliteal lymph node dissection was performed, and there was no recurrence or complication until long-term followup. This case study illustrates the importance of more detailed individual investigation of lymphatic drainage patterns from the foot to the inguinal and popliteal lymph nodes.

## 2. Case Presentation

**Figure 1:**
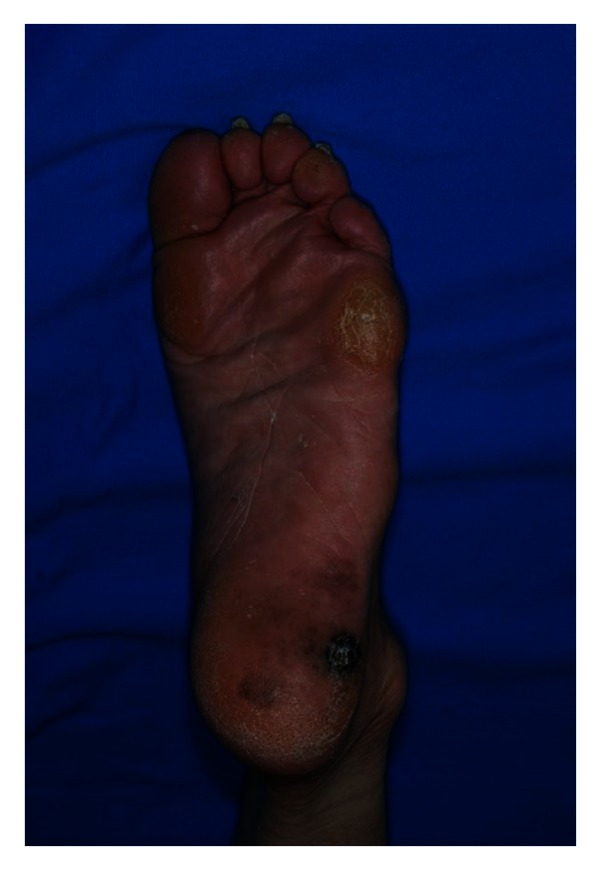
The findings of primary skin tumor when first diagnosed. The lesion was 55 × 40 mm in size with a 15 mm-diameter nonulcerated nodule in the center.

A 72-year-old man presented to the department of dermatology with a 2-week history of geographical melanotic lesion of his left heel. The lesion was 55 × 40 mm in size with a 15 mm-diameter nonulcerated nodule in the center when first diagnosed (Figure 1). The findings of dermoscopy confirmed the presence of malignant melanoma. Neither inguinal nor popliteal lymph node was palpable, and no distant metastasis or lymph node enlargement was found by contrast computerized tomography scanning and ^18^F-fluorodeoxyglucose positron emission tomography. The depth of tumor invasion was evaluated to be the superficial layer of subcutaneous fatty tissue by magnetic resonance imaging. 

**Figure 2:**
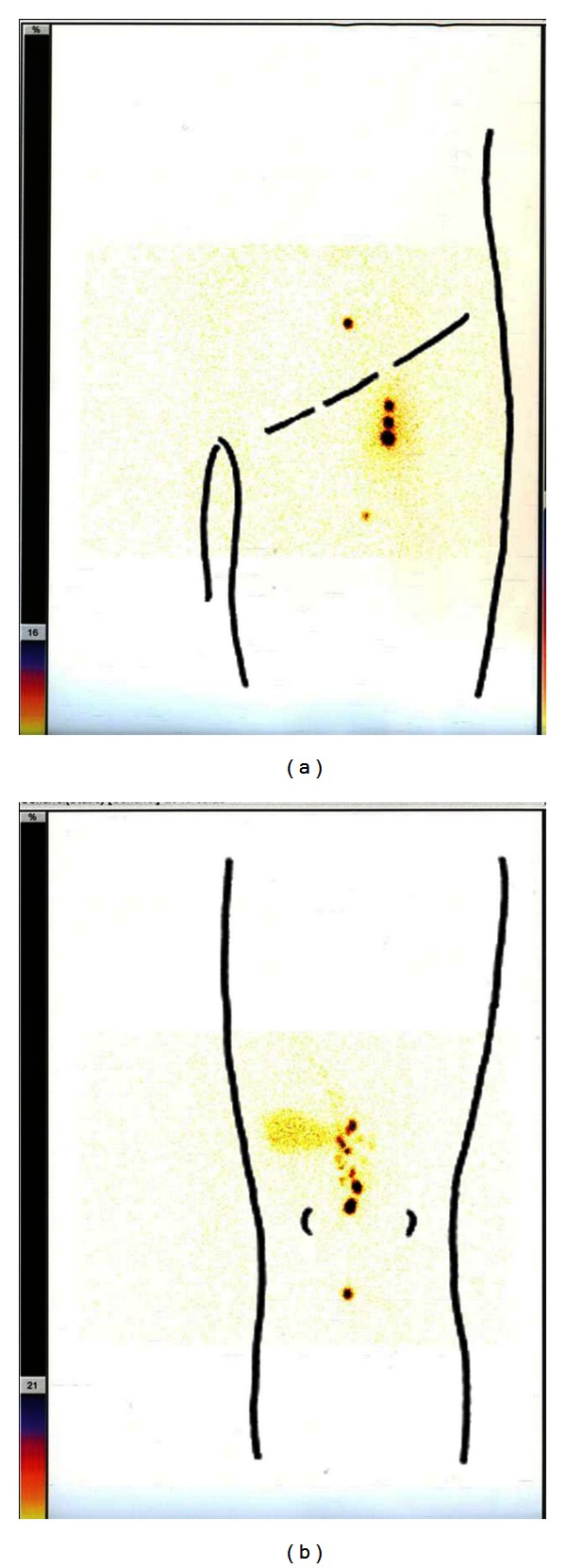
Lymphoscintigraphy on the day before surgery. Lymph nodes were detected in both (a) inguinal and (b) popliteal lesions.

The patient was referred to the department of plastic and reconstructive surgery for surgical treatment. The day before surgery, lymph nodes were detected in inguinal and popliteal lesions by lymphoscintigraphy (Figure 2). Prior to excision, 1 mL of patent blue was injected intradermally around the primary tumor. A wide local excision with 1.5 cm margin including plantar fascia was performed, along with sentinel lymph node biopsy of the inguinal and popliteal lesions using a combination of a gamma probe and the highlighted lymphatics obtained from the patent blue dye. After excision of the malignant melanoma from the left heel, the defect was covered with medial plantar flap and split-thickness skin grafting from the left femur. 

**Figure 3:**
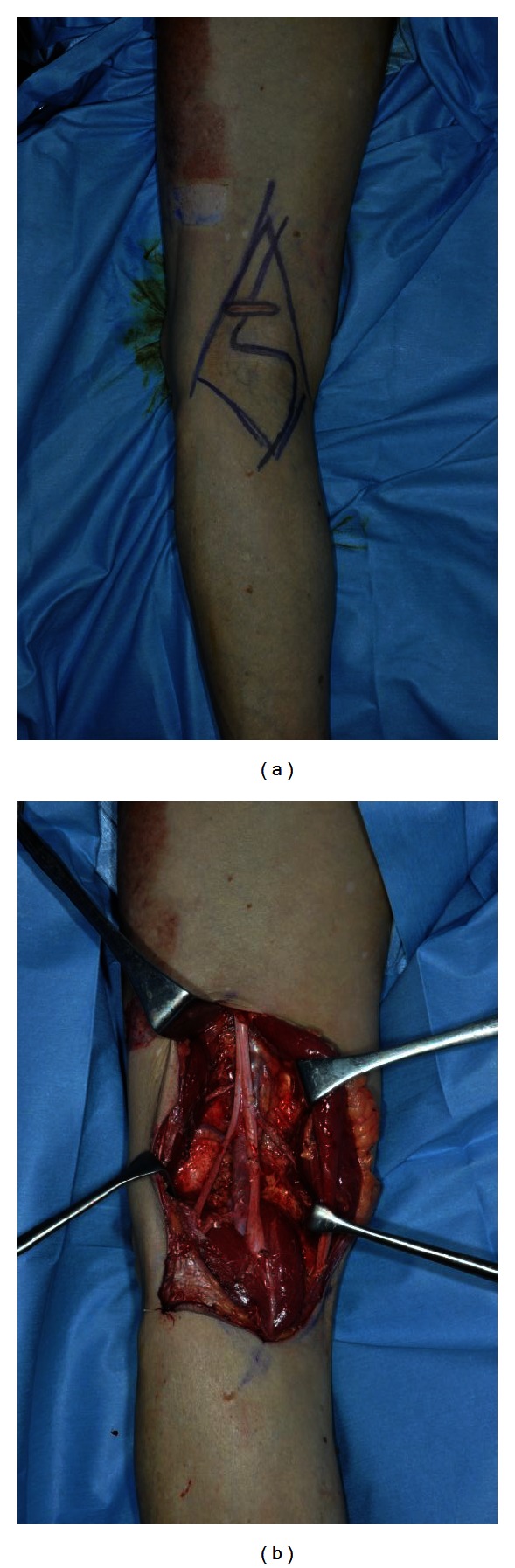
(a) The operative findings of radical popliteal lymph node dissection. A fat pad and lymphatic tissue under the popliteal fascia were dissected from around the popliteal vessels and major nerve trunks to the lower leg with an S-shaped incision. (b) Popliteal artery and vein and tibial, common peroneal, and medial sural cutaneous nerves were preserved, although the lesser saphenous vein was sacrificed.

The specimen was confirmed to be a 4.2 mm-thick, acral lentiginous melanoma, Clark's level IV, on pathologic examination. The primary site was clear of tumor at all margins; the inguinal nodes were negative (0/3), but the popliteal node was positive (1/1) for metastatic melanoma. Therefore, radical popliteal lymph node dissection was performed [1]. A fat pad and lymphatic tissue under popliteal fascia were dissected from around the popliteal vessels and major nerve trunks to the lower leg with an S-shaped incision. Although the lesser saphenous vein was sacrificed, the other vessels (popliteal artery and vein) and nerves (tibial, common peroneal, and medial sural cutaneous nerves) were preserved (Figure 3).

Examination of the specimen showed that the nodes were positive (2/2). The AJCC/UICC clinical stage was T4aN2aM0, stage IIIA. The patient went on to receive adjuvant chemoimmunotherapy with five courses of DAV-feron therapy (consisting of dacarbazine, nimustine hydrochloride, vincristine, and interferon beta) and a monthly local injection of interferon beta to the left heel and popliteal space performed by dermatologists. 

**Figure 4:**
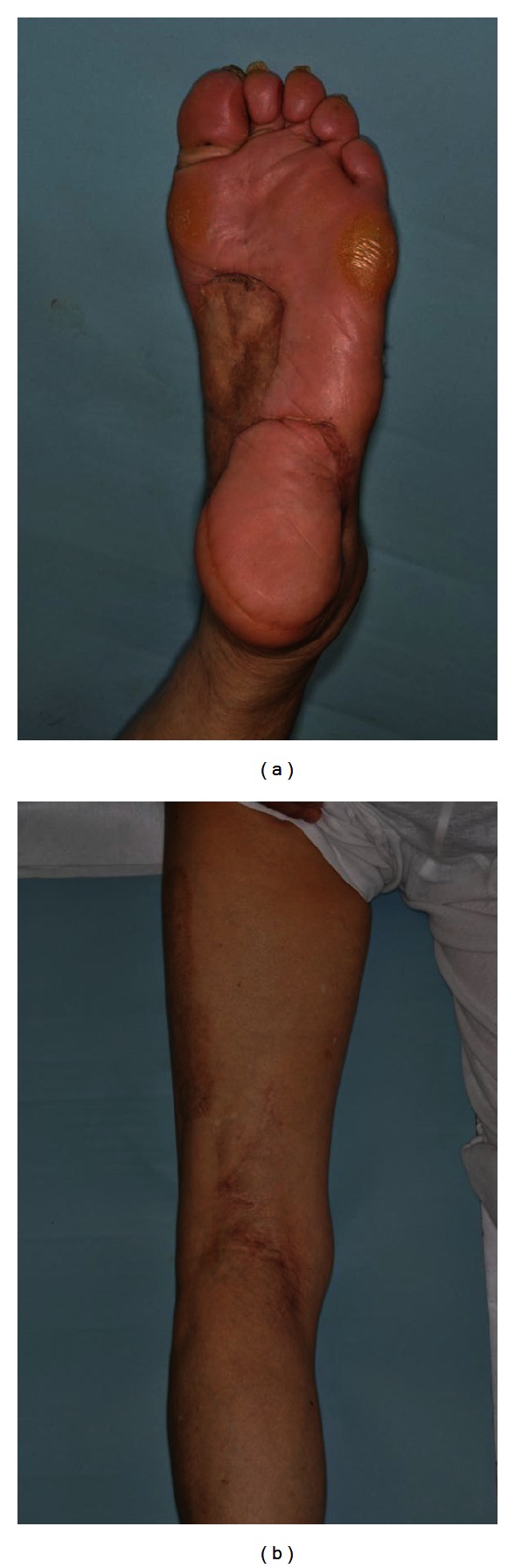
The 33-month postoperative findings of the patient's lower extremity. There were no local or systemic signs of recurrence, and lymphedema was not seen in the left lower leg.

The patient had an uneventful postoperative recovery. All wounds healed in a satisfactory fashion. There were no local or systemic signs of recurrence until his 33-month followup, and lymphedema was not seen in the left lower leg (Figure 4). 

## 3. Discussion

The most effective treatment of malignant melanoma is thought to be a surgical excision of primary tumor and metastatic lesion. The extent of excision should be necessary and sufficient from the perspective of tumor recurrence and postoperative complications, and the determination of indication for lymph node dissection is sometimes difficult. 

Lymphatic drainage of cutaneous melanoma targets cervical, axillary, or inguinal lesions in many cases, but some melanomas initially drain to unexpected areas, such as the epitrochlear region or popliteal fossa [2]. Lymph nodes in these lesions have been described as interval or in-transit nodes, including drainage to the popliteal nodes for a distal lower extremity. According to classical anatomical theory, drainage from the dorsum and medial aspect of the foot passes parallel to the course of the great saphenous vein to the inguinal lesion, and drainage from the posterolateral aspect of the heel, sole, and lateral malleolus follows the lesser saphenous vein to the popliteal legion. Thompson et al. [3] showed that positive nodes in the popliteal fossa can occur from a lesion anywhere below the knee. Biopsy of these interval nodes in melanoma indicates that they are as frequently involved with metastatic disease as the nodes in the conventional lymph node basin [4]. However, the rate of popliteal nodes detection was reported [2–8] to be from 1.8% to 9.6%, so popliteal nodal metastasis is relatively uncommon. Furthermore, the popliteal metastasis-positive and inguinal-negative rate of all melanomas located below the knee is estimated to range from 0.16% to 0.94% [2–7]. 

In these popliteal-positive and inguinal-negative cases, the issue of which lesions should be dissected is important. There is no established evidence on this subject. Because we considered popliteal nodes as a regional, not interval, lymph node basin and defined the popliteal status independent of the inguinal nodal status, only popliteal lymph node dissection without dissection of the next nodal basin was performed. As a result, good postoperative course with no recurrence or complication was achieved in our institution. Lymphedema is an unpleasant complication of inguinal and ilioinguinal lymph node dissection, which may cause a chronic feeling of heaviness, discomfort, and pain, sometimes resulting in limitation of patient activity. This is reported to occur in 9% to 55% of patients, but lymphedema is rarely seen after only popliteal lymph node dissection [9, 10]. In our case, only “interval” node dissection is thought to be an adequate treatment. Because Steen et al. reported that two popliteal node-positive and inguinal-negative cases had nodal recurrences in the groin after disease-free intervals of about 60 months [6], further long-term followup and prompt treatment for recurrence are necessary in our patient. 

Lymphoscintigraphy is now used routinely for sentinel lymph node biopsy and sometimes identifies popliteal nodal drainage, which can be the only site of nodal metastasis in some cases [4]. The first site of drainage of a lesion is the sentinel node by definition, so the popliteal node can be a sentinel lymph node, not an “interval node.” The inguinal node is not the sentinel node in all lower extremity melanomas, and all sentinel nodes identified by a lymphoscintigram should be removed. There are thought to be some patterns of lymphatic drainage from the foot to the inguinal and popliteal lymph nodes [5]. More detailed investigation of anatomical predictions of nodal drainage should be performed, and more appropriate surgical treatment is expected to improve patient's quality of life.
